# Axonal architecture of the mouse inner retina revealed by second harmonic generation

**DOI:** 10.1093/pnasnexus/pgac160

**Published:** 2022-08-16

**Authors:** Arafat Meah, Vinessia Boodram, Festa Bucinca-Cupallari, Hyungsik Lim

**Affiliations:** Department of Physics and Astronomy, Hunter College, New York, NY 10065, USA; Department of Physics and Astronomy, Hunter College, New York, NY 10065, USA; Department of Physics and Astronomy, Hunter College, New York, NY 10065, USA; The Graduate Centre of the City University of New York, New York, NY 10065, USA; Department of Physics and Astronomy, Hunter College, New York, NY 10065, USA; The Graduate Centre of the City University of New York, New York, NY 10065, USA

**Keywords:** axon, bipolar cell, amacrine cell, second harmonic generation, glaucoma

## Abstract

We describe a novel method for visualizing the network of axons in the unlabeled fresh wholemount retina. The intrinsic radiation of second harmonic generation (SHG) was utilized to visualize single axons of all major retinal neurons, i.e., photoreceptors, horizontal cells, bipolar cells, amacrine cells, and the retinal ganglion cells. The cell types of SHG+ axons were determined using transgenic GFP/YFP mice. New findings were obtained with retinal SHG imaging: Müller cells do not maintain uniformly polarized microtubules in the processes; SHG+ axons of bipolar cells terminate in the inner plexiform layer (IPL) in a subtype-specific manner; a subset of amacrine cells, presumably the axon-bearing types, emits SHG; and the axon-like neurites of amacrine cells provide a cytoskeletal scaffolding for the IPL stratification. To demonstrate the utility, retinal SHG imaging was applied to testing whether the inner retina is preserved in glaucoma, using DBA/2 mice as a model of glaucoma and DBA/2-*Gpnmb+* as the nonglaucomatous control. It was found that the morphology of the inner retina was largely intact in glaucoma and the presynaptic compartments to the retinal ganglion cells were uncompromised. It proves retinal SHG imaging as a promising technology for studying the physiological and diseased retinas in 3D.

Significance Statement Conventional fluorescence microscopy for visualizing the axons, despite numerous advantages, suffers critical drawbacks, such as low throughput and/or having to express fluorescent proteins. Here we demonstrate the use of an intrinsic radiation of second harmonic generation for imaging the axonal architecture in the unlabeled murine retina. The new technique allows insights into the retinal interneurons in health and diseases. As proof of utility, we show the persistence of the inner retina in glaucoma.

## Introduction

Imaging the retina's three-dimensional architecture is crucial for understanding the parallel processing of visual information. The neural circuit has been dissected in the physiological tissue via fluorescent labeling. Green fluorescent protein (GFP) allows targeting specific cells genetically (1–4). However, due to an expression too low for revealing thin processes, it often requires additional labeling such as Lucifer yellow or biocytin (5). Synthetic fluorophores can be microinjected to permit in situ correlation with the neurite's anatomy during electrophysiological recording, but the low throughput is ill-suited for a large-scale interrogation. Here we describe a new method to image single axon-like processes in the fresh retina without labeling. Endogenous second harmonic generation (SHG), previously demonstrated for imaging the retinal ganglion cell (RGC) axons (6, 7), can visualize all major neurons in the inner and outer retinas.

## Results

### Intrinsic SHG visualizes neurites across the inner and outer retina

**Fig. 1.**
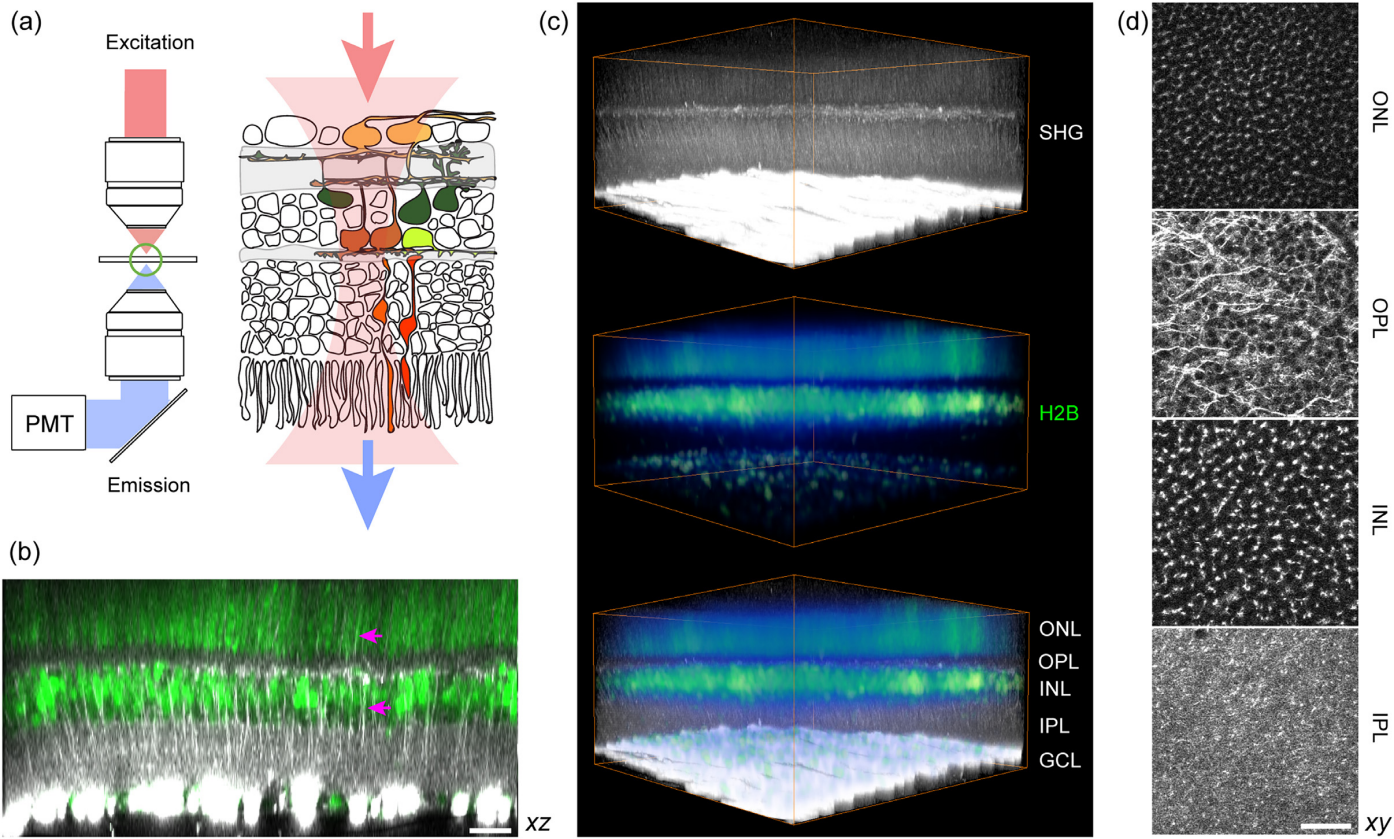
Visualizing the fresh wholemount retina by SHG. (a) Experimental configuration for detecting transmitted SHG. (b) Multiphoton imaging of the CAG-H2B-EGFP retina (10) (an axial projection of z-stack images). The plexiform and nuclear layers are segregated by SHG (gray scale) and EGFP (green), respectively. Vertical SHG processes are also visible (arrows). (c) Volumetric rendering (371 × 371 × 186 µm^3^). (d) The segmented layers (lateral projections): ONL, the outer nuclear layer; OPL, the outer plexiform layer; INL, the inner nuclear layer; and IPL, the inner plexiform layer. Scale bars, 30 µm.

SHG can be obtained from the axon because of uniformly polarized microtubules (8, 9). The nonlinear optical radiation is particularly intense from the retinal nerve fiber bundle owing to a large number of the RGC axons (6, 7). Although, in principle, SHG should also arise from the axons of non-RGC retinal cells, the signal was expected to be much weaker. Using a setup collecting the forward emission at a half of the excitation wavelength (Fig. 1a), we acquired images of the whole retina (CAG-H2B-EGFP (10)). The vertical fibers as well as neuropils were visible in the inner and outer retina (Fig. 1b). The nuclear and plexiform layers could be distinguished for 3D reconstruction (Fig. 1c) and segmentation (Fig. 1d). Major retinal neurons were recognizable, including the photoreceptors and horizontal cells in the outer retina. The emission was not autofluorescence (Fig. S1a), but exhibited the characteristics of SHG due to microtubules: It vanished when the retina was fixed with paraformaldehyde (Fig. S1b) (11). The signal was excitable from 700 to 1250 nm, unusually broad for fluorescence. Finally, it responded to a microtubule-depolymerizing agent (nocodazole) with the intensity gradually decreasing after the treatment. These results indicated that the new optical contrast is SHG due to axonal microtubules.

### Vertical SHG+ fibers of the inner retina are bipolar cell axons

**Fig. 2.**
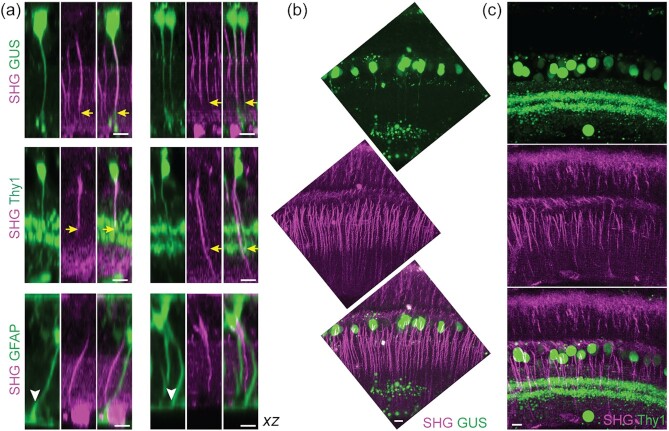
Bipolar cell axons visualized by SHG. (a) Co-registration of SHG and GFP/YFP in GUS-, Thy1-, and GFAP-GFP/YFP mice. Overlaps in GUS and Thy1 (arrows), but not in GFAP (arrowheads). (b and c) Vertical slices of the GUS and Thy1 retinas, respectively. Scale bars, 10 µm.

It seemed that the vertical SHG+ fibers of the inner retina were the axons of bipolar cells. However, Müller cells could not be ruled out as certain glial cells are known to contain polarized microtubules in their processes, e.g., oligodendrocyte (12). To resolve the cellular identity, the vertical SHG+ fibers were examined in transgenic mice expressing GFP or YFP under the neuro- and glia-specific promoters, i.e., GUS (13), Thy1 (16 line) (14), and GFAP (15).Three specific subtypes of the bipolar cell are distinguishable in the GUS-GFP retina, i.e., type 4, 7 cone and rod bipolar cells (CBC4, CBC7, and RBC, respectively) (16), whereas broad retinal neurons, including bipolar and amacrine cells, express fluorescent proteins in Thy1-YFP-16. Müller cells and astrocytes are labeled in the GFAP-GFP retina. SHG and GFP/YFP were excited simultaneously with a single 915-nm beam. The co-localization was detected in GUS and Thy1-16, but not in the GFAP retinas (Fig. 2a). Thus, it was determined that the vertical SHG processes belong to bipolar cells while Müller cells did not polarize microtubules in their processes. Comparing SHG versus GFP/YFP in the same axons revealed that the axon terminals lack SHG signals. Intriguingly, the bipolar cell axons, as seen by SHG, exhibited variable terminal depths in the IPL (Fig. 2a, arrows), which was also observable in vertical slices with a higher resolution (Fig. 2b and c).

### Classification of bipolar cell subtypes in the unlabeled fresh retina

**Fig. 3.**
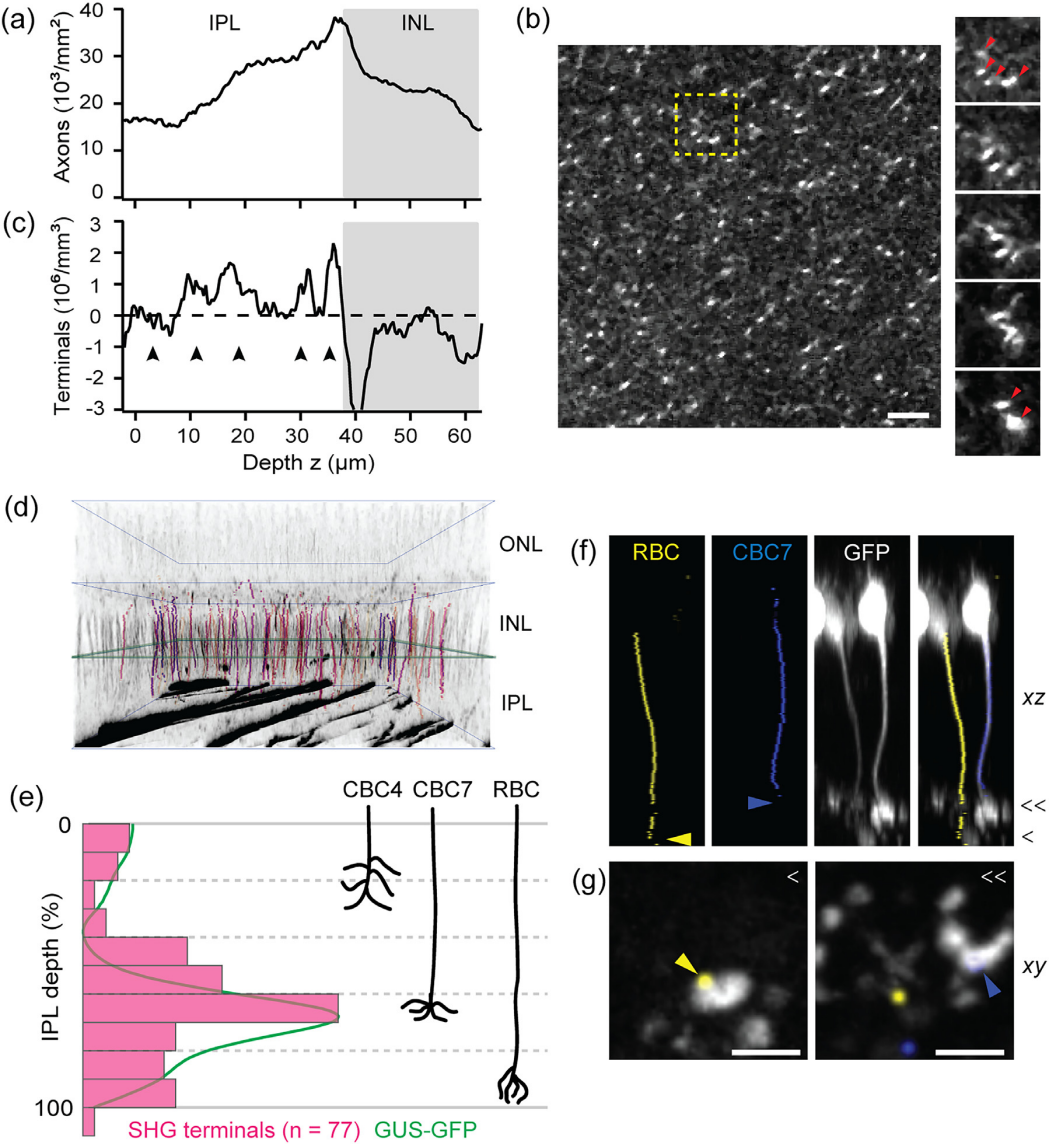
Quantification of the bipolar cell axons. (a) The density of bipolar cell axons. (b) A lateral section of the IPL. Right panels are fly-through sections corresponding to the dashed box with bipolar cell axons converging into the interstitial space of the INL (arrowheads). Scale bar, 10 µm. (c) The number of bipolar cell axon terminals, i.e., the derivative of (a), exhibits an internal structure of five layers (arrowheads). (d) 3D rendering of the GUS+ subpopulation of single SHG axons (colored and overlaid with grayscale SHG), identified by the overlap with GFP at zero IPL (green square). (e) The distribution of GUS+ SHG axon terminals (*n* = 77, magenta) and the mean GFP profile (green). Right, the depths of three GUS+ species (CBC4, 7, and RBC) for comparison. (f and g) GFP+ axon traces overlaid with GFP images, confirming the IPL depth and the characteristic morphology of RBC and CBC7 axon terminals. Scale bars, 5 µm.

Bipolar cell ramifies to an IPL sublamina to form synapses with specific amacrine cells and RGCs; therefore, the depth of the axon terminal is a chief anatomical marker of the function (17, 18). We investigated this parameter with SHG imaging. The census of bipolar cell axons was conducted (Fig. 3a). The number of axons increased steadily toward the INL and then decreased inside the INL, as some SHG fibers coalesced into the interstitial space between the cell bodies beyond the optical resolution (Fig. 3b, arrowheads). The peak density, which occurred around zero IPL depth (i.e., the interface between the IPL and INL), was approximately 40,000 to 45,000 axons/mm^2^. It was consistent with the previous counts of bipolar cells in the mouse retina (∼40,000 to 50,000 cells/mm^2^), measured by immunohistochemical staining (19, 20) or electron microscopy (21, 22). Detecting thin bipolar cell axons can be improved with a high signal-to-background ratio and a lateral resolution of SHG imaging. The derivative of the number of bipolar cell axons with respect to the depth yielded the number of axon terminals (Fig. 3c), which revealed a subdivision with four and five maxima of relatively uniform separation, suggestive of the internal structure of the IPL (arrowheads, Fig. 3c). Conceivably, the pattern mimicking the IPL subdivision emerged because of the diverse subtypes of bipolar cells. To test this idea, we examined SHG axon terminals in the GUS-GFP retinas. For an objective measurement of axon terminals, single-neurite tracing was performed on SHG z-stacks (Fig. 3d). The GUS+ subpopulation of SHG traces were identified by the superimposition with GFP at zero IPL depth (Fig. 3d). The axial distribution of GUS+ SHG axon terminals showed distinct populations of CBC4, CBC7, and RBC (Fig. 3e). Furthermore, the histogram conformed to the mean GFP profile, verifying the accuracy of measurement. The predicted cell subtypes of the GUS+ SHG axons were corroborated with GFP images (Fig. 3f and g); two cells with SHG axons ending in layers 4 and 5 (blue and yellow arrowheads, respectively) showed the characteristic morphology of axon terminals of cone and rod bipolar cells, respectively. Consequently, the SHG-based classification of bipolar cell subtypes was validated. Remarkably, it exploits only an anatomical property, which has been little utilized as a classifier. Compared to molecular classifiers revealing only select subtypes per marker, SHG accounts for all bipolar cells and is applicable to the unlabeled retina. Furthermore, free from the need for an exogenous stain (e.g., fluorescent antibodies) to penetrate thick tissue, the principle is scalable to the whole 3D retinal flatmount. The precision is limited by the axial resolution of imaging (∼1.5 µm), which is approximately 4% of the IPL thickness.

### SHG intensity varies significantly along the bipolar cell axons

**Fig. 4.**
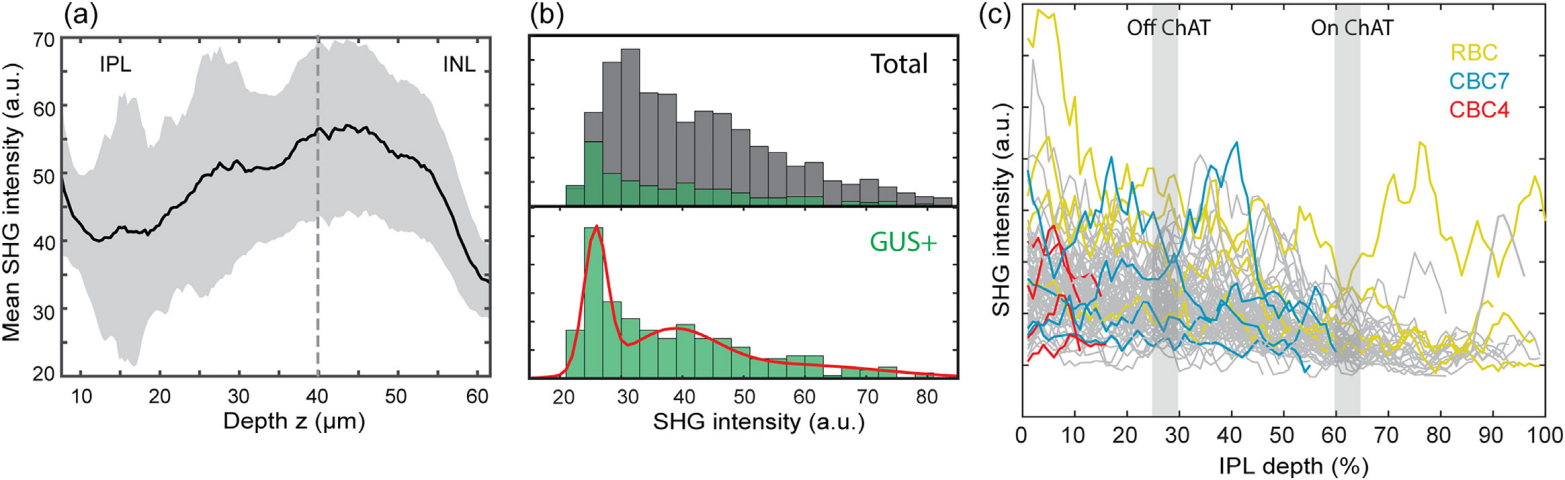
Variable SHG intensity of individual bipolar cell axons. (a) The mean SHG intensity increases toward the INL. (b) Histograms of the SHG intensity at zero IPL depth of total (gray) and GUS+ (green) bipolar cell axons. The red line shows the maximum likelihood fit to the Gaussian mixture model, which has three components. (c) The SHG intensities along the GUS+ axons (*n* = 77).

SHG intensity depends on the amount of uniformly polarized microtubules, thus scales with the axon caliber, which is of electrotonic significance but too small to measure by light microscopy. Despite significant variations, the SHG intensity of bipolar cell axons, on average, was higher close to the INL, implying the thickening of axons (Fig. 4a). The variations could stem from the subtypes of bipolar cells, e.g., OFF cells have thicker axons than ON cells. However, the SHG intensities of GUS+ axons displayed just as significant variations as total bipolar cell axons at zero IPL depth (Fig. 4b). To disentangle the variability, SHG intensity was analyzed axially along single axon traces. The individual traces showed little correlation between the SHG intensity and the IPL depth of the axon terminals. Hence, the cell type was not a major determinant of the SHG intensity. Moreover, the variations of SHG intensity were substantial along the single axons regardless of the subtype, often relative to the choline acetyltransferase (ChAT) bands (3, 23–25) (gray, Fig. 4c).

### Cytoskeletal substrate for the IPL stratification

**Fig. 5.**
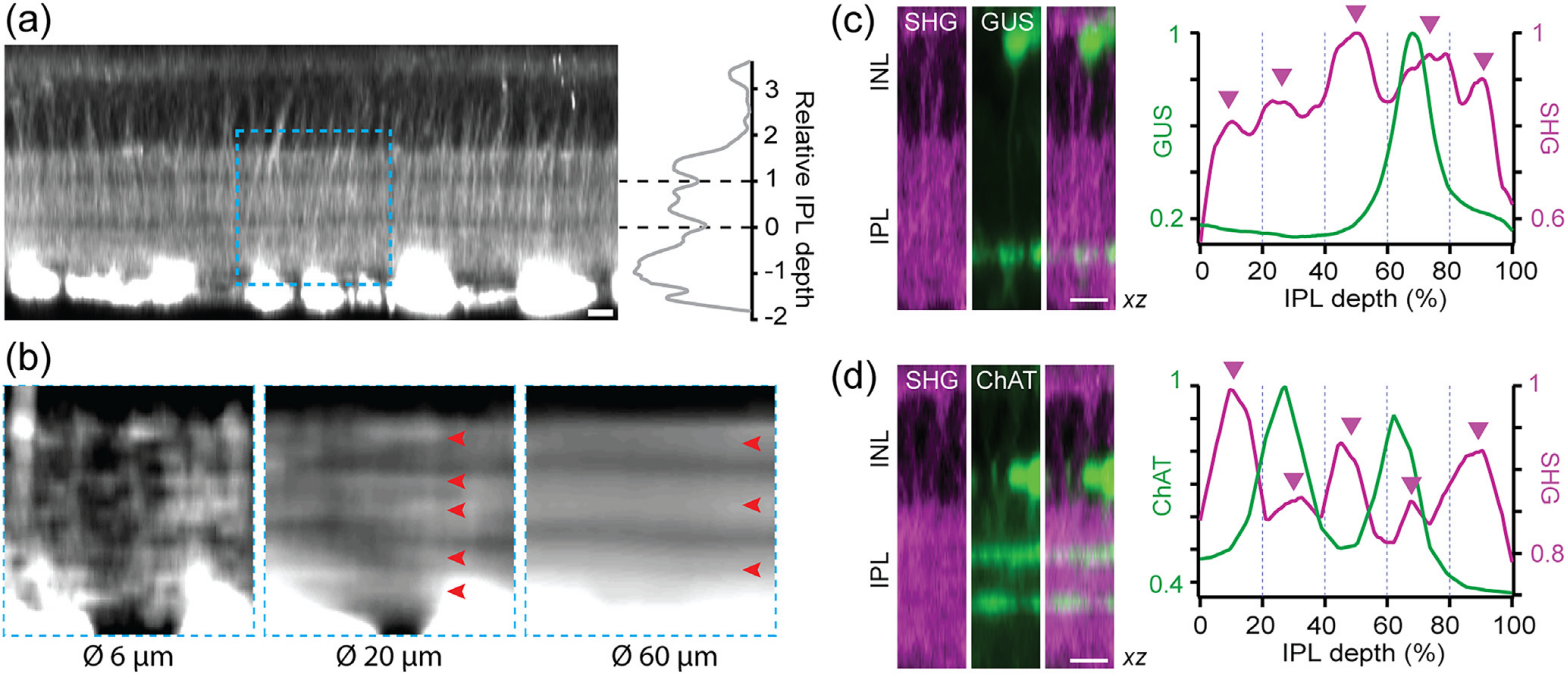
Cytoskeletal substrate underlying the IPL stratification. (a) Two intra-IPL gaps divide SHG strata approximately at the depth of ChAT bands. (b) A region, corresponding to the dashed box in (a), is averaged laterally over 6, 20, and 60 µm. (c), (d) The profiles of SHG versus GFP/YFP in the GUS-GFP and ChAT-EYFP retinas, respectively. The positions of five SHG strata are shown with arrowheads. Scale bars, 10 µm.

The IPL consists of 5 (or 10) sublaminae of equal thickness participating in discrete parallel processing (18, 26). SHG imaging of the IPL neuropil routinely revealed a subdivision with 3 strata (Fig. 5a). The axial positions of the gaps coincided approximately with the ChAT bands (Fig. 5a, right), suggesting that the SHG strata aligned with the functional sublayers of sustained ON, transient ON–OFF, and sustained OFF. Furthermore, it indicated that there exists a microtubule scaffolding underlying the IPL stratification, i.e., a cytoskeletal substrate for retinal neurons to establish proper synapses. More refined SHG strata appeared in a shorter range, i.e., approximately 5 sublayers over 20 µm (Fig. 5b), implying variable lateral frequencies of SHG+ neurites. To verify the relationship with the IPL sublaminae, SHG strata were analyzed in the GUS-GFP and ChAT-EYFP retinas. The GUS-GFP signal appeared in the fourth of 5 SHG strata (Fig. 5c), while the ChAT-EYFP bands were correctly at the 25% and 62% IPL depths relative to the SHG neuropil (Fig. 5d).

### Lateral SHG+ processes within the IPL represent a subset of amacrine cells

**Fig. 6.**
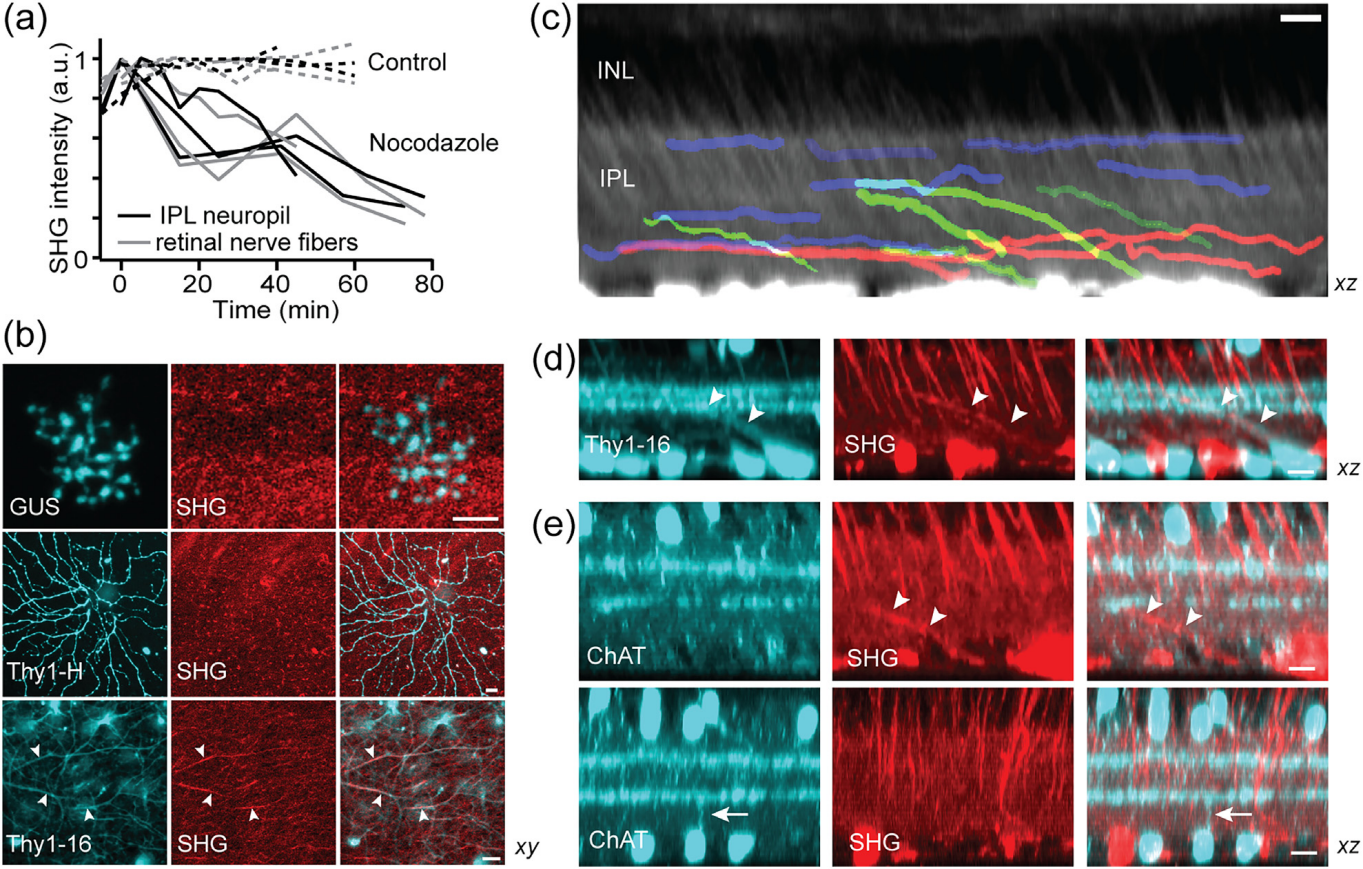
The origin of the IPL neuropil signal. (a) SHG intensity after the treatment with nocodazole. (b) Co-registration of SHG and GFP/YFP in the GUS, Thy1-YFP-H, and -16 retinas. Overlaps only in Thy1-YFP-16 (arrowheads). (c) The stratification of mid- (blue), long-range (red), and displaced amacrine cell (green) SHG+ neurites. (d and e) The identity of SHG+ displaced cell. SHG+ neurites are Thy1+ but not ChAT+ (arrowheads). Conversely, the neurites of ChAT cells are SHG- (arrow). Scale bars, 10 µm.

The molecular origin of the IPL neuropil SHG was determined as microtubules, based on the response to a nocodazole treatment where the intensity of neuropil SHG decreased at the same rate as that of the retinal nerve fibers (Fig. 6a). We asked whether the IPL neuropil SHG was due to specific types of retinal neurons. The IPL consists of three major neuronal components, i.e., RGC dendrites, amacrine cell neurites, and bipolar cell axon terminals. To find out which is responsible for neuropil SHG, we employed the transgenic retinas of Thy1-YFP (lines H and 16), where the RGC dendrites and amacrine cell neurites are labeled, respectively (14), and GUS-GFP. SHG and GFP/YFP did not co-localize in GUS-GFP and Thy1-YFP-H (Fig. 6b), excluding bipolar cell axon terminals or RGC dendritic arbors as the source. By contrast, overlaps in Thy1-YFP-16 (arrowheads) suggested that neuropil SHG originates from amacrine cell processes. The observed diffuse SHG in the IPL could result from the dense network of subresolution neurites.

As the most diverse retinal interneurons, amacrine cells perform a variety of functions in visual integration, which are dictated by the spatial range of neurites (27–29). We investigated the lateral field and axial stratification of SHG+ amacrine cells. Single-neurite tracing revealed mostly medium to wide fields (>100 µm) but scarcely narrow fields (Fig. 6c), implying that only a subset of amacrine cells gives rise to SHG. Named for the presumed absence of axons, some amacrine cells are now known to bear axon-like processes (e.g., polyaxonal amacrine cells) (28, 30–33). Plausibly, these axon-bearing amacrine cells are SHG+. In terms of stratification, SHG revealed discrete classes of amacrine cells, i.e., mono- (Fig. 6c, blue and red) and multistratified (green). The latter appeared to be displaced amacrine cells whose soma are in the ganglion cell layer. Displaced amacrine cells encompass multiple subtypes whose lateral fields range from narrow to wide (34). One of the best characterized narrow-field, and also the most numerous, displaced amacrine cells is the starburst amacrine cell (SACs) (23, 35, 36). Since SACs are not endowed with axon-like processes, we reasoned that their processes would be SHG-. To test this, we imaged the ChAT-EYFP retinas in which SACs are labeled by EYFP. The SHG+ processes of displaced amacrine cells, while Thy1+ (Fig. 6d), were not ChAT+, and vice versa (Fig. 6e, arrow and arrowheads). Together with the absence of SHG from the ChAT bands (Fig. 5), this result indicate that the SAC processes do not habor uniformly polarized microtubules.

### The broad excitability of SHG aids safer retinal imaging

**Fig. 7.**
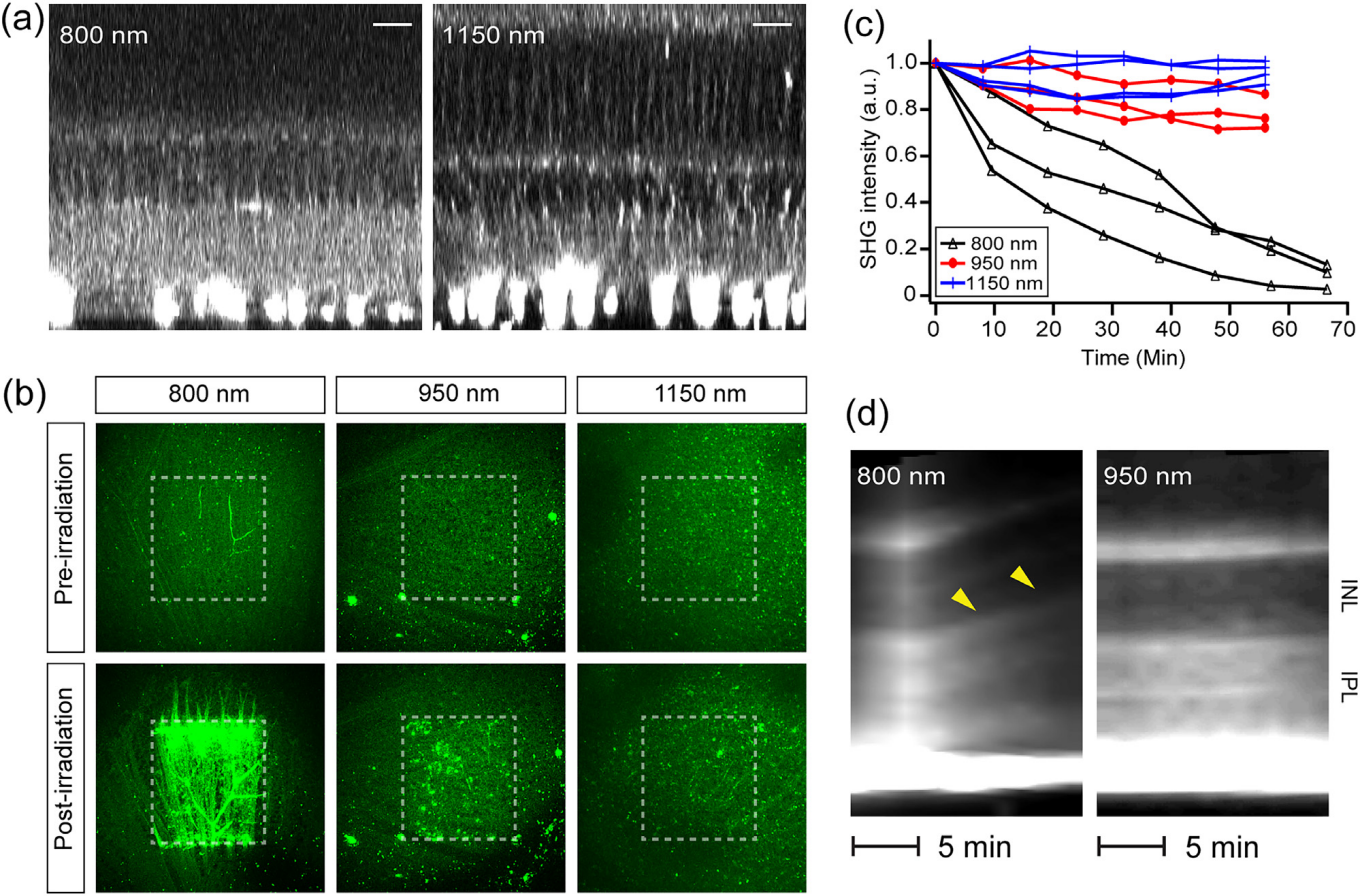
Retinal SHG imaging is tunable to longer wavelengths for safety. (a) SHG imaging at ${{\boldsymbol{\lambda }}}_{{\boldsymbol{Ex}}}$ = 800 and 1150 nm. Scale bars, 20 µm. (b and c) Light-induced changes evaluated by autofluorescence and SHG, respectively. The postirradiation images are the 10^th^ of z-stacks acquired every 5 min (i.e., *t* = 50 min). (d) SHG kymographs of the retina under continuous illumination. Axial swelling at 800 nm (arrowheads) but not at 950 nm.

Since imaging non-RGC cells required an excitation about more than five times higher than for the RGC axons, the risk of photodamage was elevated. There are measures to improve photosafety. First, the laser beam could be switched off during the fly-back motion of the galvoscanner, i.e., when images are not acquired, reducing the average power that the sample receives by approximately 37% with no adverse effect on the image quality. In addition, the excitation could be tuned to a longer near-infrared wavelength, which is in general safer than shorter wavelengths (37, 38). Unlike fluorescence, SHG can be excited at any wavelength since it does not hinge on molecular absorption and indeed, similar image qualities were obtained from 700 to 1250 nm (Fig. 7a). To assay the photosafety of SHG imaging, regions in the fresh retina were irradiated at 800, 950, and 1150 nm at the same energy density ( 500 J/cm^2^) and then evaluated by autofluorescence and SHG. The photodamages were negligible at 1150 nm compared to 800 nm (Fig. 7b). Also, the SHG intensity from the ganglion cell layers reduced significantly under the continuous illumination at 800 nm, indicating the loss of cytoskeleton, while it was relatively maintained at 950 and 1150 nm (Fig. 7c). Another indicator of photodamage was provided by the swelling of the retina, which is presumably a result of disrupted transcellular water distribution by Müller cells due to excessive K^+^ current (39). The threshold light dosage to initiate the expansion depended on the wavelength, which was much higher at 950 nm than at 800 nm (Fig. 7d). The effect was not noticeable at 1150 nm, where almost no visual transduction was evoked, establishing the relative safety of longer excitation wavelengths.

### The persistence of the inner retina in glaucoma

**Fig. 8.**
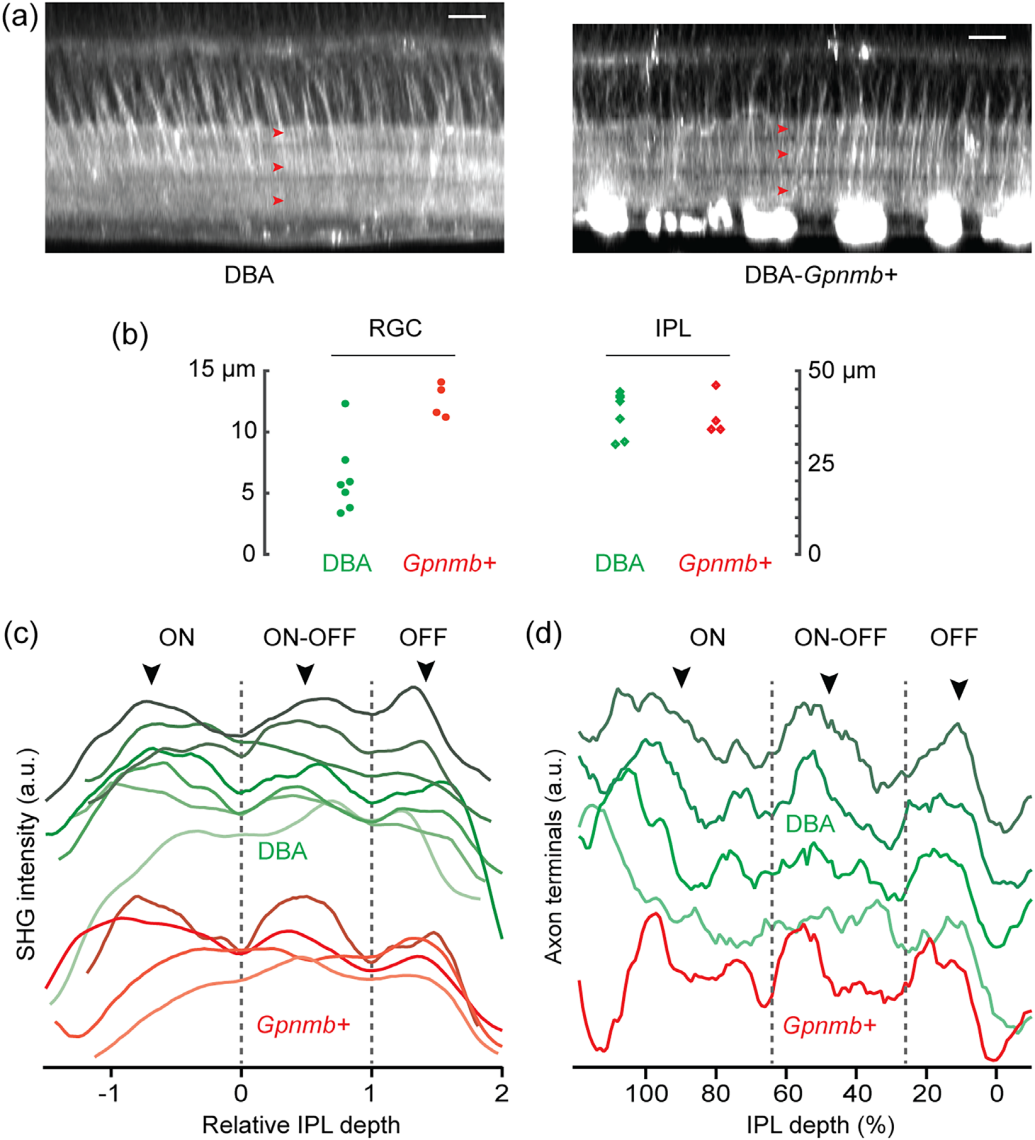
Morphological changes in the glaucomatous inner retina. (a) Representative SHG images of DBA and DBA-*Gpnmb+*. The IPL sublaminae appear normal in both strains (arrowheads). Scale bars, 20 µm. (b) The thicknesses of the RGC axon bundles vs. the IPL. (c) The average SHG intensity of the IPL neuropil. (d) The density of axon terminals of bipolar cells.

To prove the utility, retinal SHG imaging was recruited for asking an outstanding question in glaucoma; namely, whether the pathology is restricted to the RGCs or also involves non-RGC parts of the inner retina. In addition to the RGC dendrites and synapses that degrade in early glaucoma (40–45), the presynaptic components of amacrine cells and bipolar cell axons might be impaired. The notion has been tested by various modalities in primates and rodents, but the results have been inconsistent (46–49). We evaluated the inner retina of a mouse model of glaucoma DBA (50, 51) and the nonglaucomatous control DBA-*Gpnmb+* (52) (*n*  = 7 and 4 retinas, respectively, 13 to 16 months old). Regions around the central retina (0.5 mm from the optic nerve head) were imaged at an excitation wavelength of 950 nm. The SHG images of DBA retinas revealed that, despite the substantial loss of the RGC axons, the morphology of the IPL was largely intact (Fig. 8a and b). Also, the SHG intensity in the IPL was comparable to that of DBA-*Gpnmb+*, suggesting that the axon-like neurites of amacrine cells were preserved. Our results complement the previous immunohistochemistry data confirming the structural integrity of amacrine cells in glaucoma (53–56).

Evidence suggests the progression of glaucoma is circuit-dependent; especially, the ON and OFF pathways exhibit divergent susceptibilities at the level of the RGCs (57–60). The differential susceptibility could be RGC-autonomous or pervade the larger inner retina beyond the RGCs. We decided to illuminate this outstanding question in a new light with SHG. First, the IPL sublaminae of DBA and DBA-*Gpnmb+*were analyzed for differential degeneration. The previous study has found, by counting the immunostained cell bodies, that specific amacrine cells are compromised in glaucoma (61). Should the neurites of certain amacrine cells stratifying ON or OFF layers be lost substantially, we reasoned, it will lead to a relative thinning of the IPL sublaminae, which should be detectable by SHG imaging regardless of whether the amacrine cells themselves are visualized by the nonlinear optical signal. We found three SHG strata maintained relatively normal thicknesses in DBA (Fig. 8c, arrowheads), indicating the resilience of sustained ON, transient ON−OFF, and sustained OFF layers in glaucoma.

Next, we studied the integrity of ON vs. OFF bipolar cell axons following glaucoma-like injury. Immunostaining against Sty2, which labels the axon terminals of type 2 and 6 bipolar cells (20), has verified the preservation of type 6 ON bipolar cell axons but OFF axons remain obscure (62). We distinguished the ON and OFF bipolar cells by the IPL depth of axon terminals, quantifying the entire population in the same wholemounts. The density of axon terminals showed distinct populations of the ON, ON−OFF, and OFF cells (Fig. 8d, arrowheads), indicating all the subtypes were largely intact. Taken together, SHG imaging data verified the persistence of the non-RGC axons of the inner retina in glaucoma and no morphological disparity was found between the ON and OFF pathways.

## Discussion

### SHG as a phenotype of axon-like neurites

SHG is shown to visualize the axons of all five major classes of retinal neurons. New properties are discovered with SHG, such as the fluctuating diameters of bipolar axons. Since the morphology of the axon controls the frequency response so is key for sculpting the visual signals, e.g., the shorter OFF bipolar axons are more suitable for conveying fast electric transients (18), the modulated axon caliber is likely to have consequences for visual processing rather than being a random feature. For example, it could be a mechanism to fine-tune the relative timing between parallel visual pathways of remote vertical integrations or the IPL sublayers. Another finding obtained with SHG is regarding the neurites of amacrine cells, which are the most diverse among retinal neurons reflecting the multitude of their functions in the visual processing. New subtypes of amacrine cells have been discovered with emerging techniques (1, 4, 32, 63, 64), but the taxonomy may be still incomplete. SHG is obtained only from a certain group of amacrine cells, presumably axon-bearing kinds comprising as much as 30% of amacrine cells in the mouse retina (1, 30, 32). The axon-like properties of amacrine cell neurites include morphology, spatial range, electrical spiking (33), and immunoreactivity against phosphorylated neurofilament-H (pNF-H) (65). Our study raises microtubule polarity as another axonal phenotype, adding a new dimension to the classification of amacrine cells (66). It opens a set of new questions, e.g., whether all axon-bearing amacrine cells are SHG+; whether the uniform polarity is maintained across the entire length of neurites; and how SHG correlates with other axon-like phenotypes such as pNF-H immunoreactivity.

### Action at a distance in retinal neurodegeneration

To determine the precise loci of pathogenesis is a prerequisite to proper understanding and developing therapeutic strategies of neurodegeneration. Using SHG imaging, we investigated the sites displaying asymmetric susceptibility of ON and OFF pathways in glaucoma, i.e., whether it is confined to RGCs or extends to a larger inner retina. Considering the origin of dichotomy in the outer retina (67, 68), it seemed plausible that the intermediate bipolar cells connecting the inner and outer retinas might be involved. However, our retinal SHG imaging did not yield any evidence that either bipolar or amacrine cell axons are lost in the mouse model. Intact amacrine cells may be crucial for glaucoma therapy, not only for preserving the cytoskeletal scaffolding of the IPL but also for maintaining amacrine cell-derived signals required for the growth of the RGC axons (69). Surveying a large 3D retina without fixation or sectioning, as demonstrated in this study, can be useful for unraveling the relationship between distant cells in other retinal degeneration, e.g., retinitis pigmentosa (70), where significant remodeling of the inner retina follows after the loss of photoreceptors (71).

### Comparison to other techniques

Two-photon excited fluorescence microscopy is increasingly common in the vision research (35, 72–78). In contrast, SHG microscopy, which has been widely used for structural imaging of collagen and myosin, is extended to the central nervous system only recently (79). Retinal SHG imaging can be performed using the same setup as two-photon fluorescence microscopy with minimum alterations, e.g., the forward signal detection. Along with the usual benefits of light microscopy, SHG brings new advantages to retinal imaging. As an intrinsic contrast, it is available from most species from teleost to mammals thus can facilitate comparative studies without transgenic animals. In contrast to other intrinsic contrasts such as reflectance (80–82) or autofluorescence (74, 75), SHG visualizes the IPL subdivision and the vertical fibers, which are salient features of visual processing. Furthermore, the relatively specific molecular origin makes the data interpretation straightforward.

## Materials and methods

### Animals

All procedures were approved by the Hunter College Institutional Animal Care and Use Committee (IACUC). All mice were obtained from The Jackson Laboratory and housed in the animal facility at Hunter College: CAG-H2B-EGFP (B6.Cg-Tg(HIST1H2BB/EGFP)1 Pa/J, #006,069), GUS-GFP (Tg(Gnat3-GFP)1Rfm/ChowJ, #026,704), Thy1-YFP-H (B6.Cg-Tg(Thy1-YFP)HJrs/J, #003,782), Thy1-YFP-16 (B6.Cg-Tg(Thy1-YFP)16Jrs/J, #003,709), GFAP-GFP (FVB/N-Tg(GFAPGFP)14Mes/J, #003,257), DBA (DBA/2 J, #000,671), and DBA-*Gpnmb+* (DBA/2J-*Gpnmb+*/SjJ, #007,048). ChAT-EYFP mouse was obtained by crossing ChAT-IRES-Cre knock-in (B6.129S-*Chat*^*tm1(cre)Lowl*^/MwarJ (#031,661) (83) with R26R-EYFP reporter mice (B6.129 × 1-*Gt(ROSA)26Sor*^*tm1(EYFP)Cos*^/J, #006,148) (84).

### Tissue preparation and pharmacology

The retinal wholemounts were prepared as previously described (6, 7). Animal was euthanized by CO_2_ inhalation and the eye was enucleated. The retinal wholemounts were immersed in the oxygenated Ames’ medium for imaging. It took less than 30 min from animal's euthanasia to the beginning of imaging. For vertical slices, the fresh retinal wholemount was embedded in 6% agar and cut into 250-µm slices with a vibrating blade microtome (Leica VT100 S). For nocodazole treatment, the drug was added to the Ames’ medium at the final concentration of 33 µM.

### SHG microscopy

SHG microscopy was performed using a setup previously described (6, 7). Short pulses of a 150-fs duration and an 80-MHz repetition rate from a Ti: Sapphire laser tunable from 700 to 1050 nm (Chameleon; Coherent, Inc.) or an optical parametric oscillator tunable from 1050 to 1250 nm (OPO, Angewandte Physik & Elektronik GmbH) were used for excitation. The polarization state of excitation beam was controlled with half- and quarter-waveplates. A Pockels cell (Conoptics 350–80LA) was inserted in the beam path for switching off the laser beam during the fly-back motion of galvoscanner (“fly-back blanking”), which was driven with an electrical signal synchronized with the galvoscanner. The excitation beam was focused with a water-dipping objective lens (Nikon CFI75 16 × 0.8NA or Leica HC FLUOTAR L 25 × 0.95NA). The average power was 100-150 mW at the sample. The forward-propagating SHG signal was collected with a setup comprised of an objective lens (Olympus UApo340 40 × 1.35NA), a narrow-bandpass filter (<20-nm bandwidth) with the center wavelength at a half of the excitation wavelength, and a photomultiplier tube (PMT; Hamamatsu H10770PA-40). For an efficient collection of SHG signal, the collection objective lens had a high NA and a high ultraviolet to blue transmission. It was co-axially aligned, but did not have to be strictly confocal, with the focusing objective lens. Images were acquired with 512 × 512 pixels. The pixel dwell time was approximately 3 µs. Typically 1 to 5 frames were acquired at the frame rate of ∼1.5 Hz. The z step of z-stacks images was 2 or 3 µm.

### Image processing and data analysis

Image processing was done using ImageJ (85) and MATLAB (MathWorks, Inc.). 3D reconstruction was obtained with Amira (Thermo Scientific). Segmentation was done using Trainable Weka Segmentation plugin (86). Single-neurite tracing was performed by an automatic procedure modified from single-particle tracking (87, 88) or semiautomatically by ridge detection (89, 90).

## Supplementary Material

pgac160_Supplemental_File

## Data Availability

All data are included in the manuscript and/or supporting information.
