# Supplementary material for: Axonal architecture of the mouse inner retina revealed by second harmonic generation
Source: PNAS Nexus. 2022 Aug 16;1(4):pgac160. doi: 10.1093/pnasnexus/pgac160 (PMC9463061; doi:10.1093/pnasnexus/pgac160)
Supplement: pgac160_Supplemental_File [file pgac160_supplemental_file.docx]

**
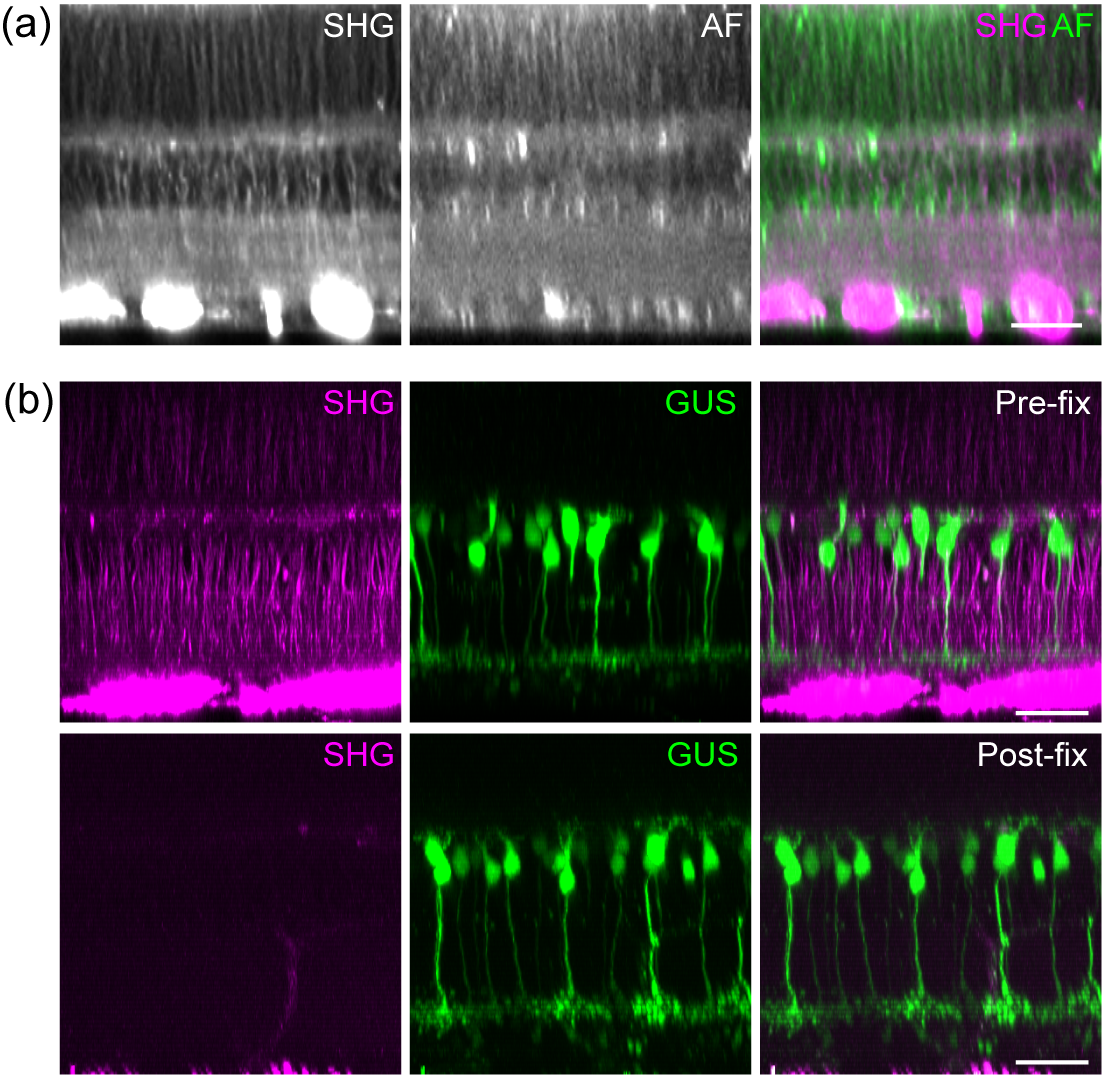
**

**Figure S1**. The new optical signal from the retina is SHG. (a) SHG and autofluorescence (AF) from the fresh retinal wholemount. (b) SHG, but not GFP, was lost after paraformaldehyde fixation of the GUS-GFP retina. Scale bars, 30 µm.
